# Longitudinal Development of Health-related Quality of Life and Fatigue in Children on Home Parenteral Nutrition

**DOI:** 10.1097/MPG.0000000000003329

**Published:** 2021-10-22

**Authors:** Sjoerd C.J. Nagelkerke, Hedy A. van Oers, Lotte Haverman, Lotte E. Vlug, Barbara A.E. de Koning, Marc A. Benninga, Merit M. Tabbers

**Affiliations:** ∗ Amsterdam UMC, University of Amsterdam, Emma Children's Hospital, Pediatric Gastroenterology, Amsterdam Gastroenterology, Endocrinology and Metabolism, Amsterdam Reproduction & Development; † Emma Children's Hospital, Amsterdam UMC, University of Amsterdam, Child and Adolescent Psychiatry & Psychosocial Care, Amsterdam Reproduction and Development, Amsterdam Public Health, Amsterdam; ‡ Erasmus University Medical Center, Sophia Children's Hospital, Pediatric Gastroenterology, Rotterdam, The Netherlands.

**Keywords:** chronic intestinal failure, fatigue, health-related quality of life, longitudinal, pediatric

## Abstract

**Objectives:**

The aim of the study was to describe the longitudinal development of health-related quality of life (HRQOL) and fatigue in children with chronic intestinal failure (CIF) on home parenteral nutrition (PN) and compare these children to the general population.

**Methods:**

Prospective, observational study conducted over 7 years in patients suffering from CIF receiving home PN from 2 tertiary hospitals in the Netherlands. Every 6 months, parents (if child <8 years old) or patients (if child ≥8 years old) completed 2 questionnaires: Pediatric Quality of Life Inventory 4.0 (PedsQL) Generic and Fatigue on the KLIK (kwaliteit van leven in kaart [Dutch Acronym for Quality of Life in Clinical Practice]) Patient Reported Outcome Measures portal, which were compared with the general population. Linear mixed models (LMMs) were constructed to investigate the course of HRQOL over time.

**Results:**

Thirty-five patients were included (40% girls). At time of last KLIK contact, patients received HPN for a median of 5.3 years (interquartile range [IQR]: 2.9–9.7). In total, 272 questionnaires were completed. PedsQL generic total score for ages 5 to 7 and 8 to 12 years was significantly lower than the general population (*P* < 0.01 for both age groups) with effect sizes of 0.73 and 0.71, respectively. PedsQL fatigue total score for ages 5 to 7 years was also significantly lower (*P* = 0.01; effect size 0.70). LMMs for PedsQL Generic and Fatigue total score 2 to 7 and 8 to 18 years showed no significant coefficient for duration of home PN.

**Conclusions:**

Children suffering from CIF receiving home PN ages 5 to 12 years report lower HRQOL scores than the general population. HRQOL and fatigue do not change during long-term treatment with home PN in these children.

**What Is Known**

- Children suffering from chronic intestinal failure who receive home parenteral nutrition are at risk of decreased health-related quality of life and fatigue as reported by cross-sectional studies.
- Survival is increasing in patients suffering from chronic intestinal failure meaning more focus on long-term outcomes is vital.
**What Is New**
- Children ages 5 to 12 years with chronic intestinal failure report lower health-related quality of life and greater fatigue than the general population.
- Health-related quality of life and fatigue do not change during long-term treatment with home parenteral nutrition in children with chronic intestinal failure.

In children, intestinal failure (IF) is characterized by the inability of the gut to digest and absorb the required fluids and nutrients for adequate growth and homeostasis. Children with IF are (partly) dependent on parenteral nutrition (PN) to meet their daily required intake of fluids and nutrients (1). IF can be the result of a variety of conditions, such as short bowel syndrome, motility disorders, and congenital enteropathies (1). Children with chronic IF require PN for at least 3 months. These children often receive their PN at home (2). This complex and time-consuming treatment puts social and psychological pressure upon the child and parents (3). We previously showed that for parents, home PN treatment of their child is stressful and influences daily functioning (4).

In the past two decades, survival rates for children with chronic IF on home PN are rising because of improvements in neonatal and surgical care, multidisciplinary care, and improvements in the management of complications associated with chronic PN use (5,6). As a consequence, researchers and physicians are now also focusing on other long-term essential outcomes, such as neurodevelopmental outcomes and health-related quality of life (HRQOL) (7,8).

A recent review summarized 8 cross-sectional studies reporting on HRQOL among a total of 264 children suffering from IF. They showed that these children frequently suffer from significantly impaired physical, social, and emotional functioning when compared with healthy controls or reference data (7).

A recent cross-sectional study showed that children suffering from IF showed greater fatigue and lower levels of physical activity and function when compared with healthy children (9). It is not known whether HRQOL and fatigue change over time in children with chronic IF. As children with IF are becoming older and reach adulthood, it is important to explore the course of HRQOL and fatigue over time. It is our hypothesis that HRQOL and fatigue improve over time in these patients. Therefore, the aims of this study are to describe HRQOL and fatigue in children with CIF and compare these children to the general population, to describe HRQOL and fatigue over time in children with CIF on home PN, and to identify variables associated with impaired HRQOL and fatigue in children with CIF.

## METHODS

### Study Design and Ethics

This was a multicenter, prospective, observational study conducted in the pediatric intestinal failure units of the Emma Children's Hospital/Amsterdam UMC and the Sophia Children's Hospital/Erasmus Medical Centre. The ethical review board of the Academic Medical Center Amsterdam declared that this study did not have to be reviewed by a medical ethics board according to Dutch law on medical research with humans (WMO).

Data from the KLIK (kwaliteit van leven in kaart [Dutch Acronym for Quality of Life in Clinical Practice]) Patient Reported Outcome Measures (PROM) portal and patient records were used. Parents and patients (≥8 years) were invited with a letter from the home PN team to register online on the KLIK website (*www.hetklikt.nu*) as part of standard care. Parents and patients ≥8 years were asked to complete PROMs (questionnaires) about relevant outcomes before their outpatient consultation. The answers were converted in an online KLIK PROfile and discussed during consultation with the healthcare professional (10). PROMs regarding HRQOL and fatigue were used for this study if online informed consent was given. At the age of 18, the use of the KLIK PROM portal stopped as patients were transitioned to adult care. Furthermore, if patients weaned from PN, the use of the KLIK PROM portal stopped as patients were no longer cared for by the home PN team.

### Patients

All families of children (0–18 years of age) who suffered from chronic IF and received home PN for ≥3 months from the IF unit of one of the centers were invited to use KLIK in daily clinical care. From June 2012 onwards, patients with chronic IF receiving home PN treated at the Emma Children's Hospital/Amsterdam UMC were introduced to KLIK and from August 2014, patients treated at Sophia Children's Hospital/Erasmus Medical Centre were introduced. Patients were followed until February 2020. Centers both followed similar therapeutic approaches with regards to children on home PN and had comparable weaning strategies (where oral foods are preferred over supplementary tube feeding) in accordance with the latest guidelines on home PN (2,11).

### Measurements

#### Medical Characteristics at the Start of Home Parenteral Nutrition

Information regarding the disease history was recorded from the child's medical charts. Collected data included: date of birth, sex, type of underlying disease leading to chronic IF, bowel characteristics (such as the length of remaining small bowel), gestational age, whether the patient was small for gestational age (birth weight ≤2SD), age at start of PN and age at discharge with home PN. Furthermore, information concerning the hospitalization before the start of home PN was recorded including the number of surgical procedures, number of septic episodes, number of days spent at intensive care unit (ICU) and number of days spent at the ward before discharge with home PN.

#### Medical Characteristics at Time of Each Completed Questionnaire

For each completed questionnaire, the following characteristics were recorded from the medical charts: age at completion of questionnaire and data regarding the number of nights patients received PN were recorded.

### Questionnaires

HRQOL of the child was assessed with the validated generic Pediatric Quality of Life Inventory 4.0 (PedsQL). Fatigue was assessed with the validated PedsQL fatigue. All questionnaires were distributed every 6 months. Information regarding liability, validity, and normative data can be found in Supplemental Digital Content 1 (12–18).

### Statistical Analysis

Sociodemographic and medical characteristics were presented using appropriate descriptive statistics depending on normality.

(Aim 1) Each separate questionnaire was compared to the Dutch general population. If a patient or parent had completed multiple questionnaires, the most recently completed questionnaire was used to compare with normative data. Comparability of children suffering from chronic IF and the general population was assessed for the PedsQL generic by comparing age and patient sex between groups using Mann-Whitney *U* test and Fisher exact test, respectively. For PedsQL fatigue, this data was not available.

PedsQL generic core scales for each age group were compared with the Dutch general population using the Mann-Whitney *U* test (15,16).

One sample *t* test was used to compare the PedsQL fatigue to previously reported normative values of the Dutch general population, all assumptions for the 1 sample *t*-test were met (17). Cohen d was calculated to assess the effect size of the 1 sample *t*-test.

Effect size of 0.20 ≤ 0.49 was considered small, 0.50 ≤ 0.79 was considered medium and ≥0.80 was considered large.

(Aims 2 and 3) To evaluate the course of HRQOL over time and to evaluate the association between HRQOL and several patient characteristics, linear mixed models (LMMs) were used. LMMs account for correlations in repeated measures per subject and allow measurements to be taken at different time points per subject to model the longitudinal course of HRQOL. Furthermore, linear mixed models are robust for missing at random missing data points.

For each completed questionnaire, the standard deviation from the mean of the general population was calculated. An LMM was built for total scores of both the PedsQL generic age 2 to 7 and age 8 to 18 as well as the PedsQL fatigue age 2 to 7 and age 8 to 18. The fixed effects that were included were as follows: duration of home PN, prematurity (born <37 weeks of gestation), underlying diagnosis, and number of nights of PN infusions per week. Random intercepts were included in the random effects part of the model; the optimal random effect structure was selected using the Akaike information criterion where lower scores indicate a better fit. A restricted maximum likelihood was used as an estimation method. To evaluate whether each linear mixed model met its assumptions, normal distribution of the models residuals was investigated by inspecting a histogram and homoscedasticity of the residuals was investigated by plotting the residuals against the dependent variable (19). Evaluation of the association of other patient characteristics on HRQOL was not possible because of violation of these assumptions.

## RESULTS

### Patients

Of the 51 patients who were asked to use their KLIK data for this study, 35 agreed to participate and were included (participation rate of 69%). Fourteen patients were girls (40%), 30 patients (86%) were treated in the Emma Children's Hospital, 5 (14%) were treated in the Sophia Children's Hospital. Clinical characteristics are displayed in Table 1. Fifteen patients were born preterm (43%), median gestational age was 38 weeks (interquartile range [IQR]: 34.6–39.8), 3 patients (9%) were small for gestational age (<−2SD). Fifteen children suffered from short bowel syndrome (43%), 12 from motility disorders (35%), 4 from a congenital enteropathy (11%), and 4 from other diseases leading to chronic IF (11%). During data collection, 11 patients weaned from PN and ceased usage of the KLIK PROM portal as treatment by the home PN team stopped, median time between last KLIK contact and home PN cessation was 1.5 months (IQR: 1.0–2.5).

**TABLE 1:** Clinical characteristics of 35 included patients

Sex, female	14 (40)
Gestational age, weeks	38.0 (34.6–39.8)
Small for gestational age	3 (9)
Age at start home PN, weeks	30 (17–302)
Age at last KLIK PROM completion, years	7.9 (5.5–13.0)
Home PN duration at last KLIK PROM completion, years	5.3 (2.9–9.7)
Underlying disease	
Short bowel syndrome	15 (43)
Length of small bowel after first surgery, cm	28 (20–120)
Type 1 short bowel syndrome (end jejunostomy)	1 (7)
Type 2 short bowel syndrome (jejunocolic anastamosis)	9 (60)
Type 3 short bowel syndrome (jejunoileal anastamosis)	5 (33)
Intestinal neuromuscular motility disorder	12 (34)
Congenital enteropathy	4 (11)
Other	4 (11)
Other chronic condition	16 (46)
Number of nights PN (days per week)	7 (6–7)
Number of nights PN containing lipids (days per week)	4 (2–7)
Time spent on ICU prior discharge with home PN (days)	6 (0–18)
Time spent on ward prior discharge with home PN (days)	76 (25–114)
Number of surgical procedures prior discharge with home PN	2 (1–4)
Number of septic episodes prior discharge with home PN	1 (0–1)

All continuous data are presented as median (interquartile range), all categorical data are presented as n (%). PN = parenteral nutrition, KLIK = kwaliteit van leven in kaart (Dutch Acronym for Quality of Life in Clinical Practice), PROM = patient reported outcome measure, ICU = intensive care unit.

At time of the last KLIK contact, patients were median of 7.9 years of age (IQR: 5.5–13.0) and received home PN for a median of 5.3 years (IQR: 2.9–9.7). At time of the last KLIK contact, patients received PN for a median of 7 nights a week (IQR: 6–7) and intravenous lipids were received for 4 nights a week (IQR: 2–7).

### Health-related Quality of Life

In total, 272 questionnaires were completed, 168 PedsQL generic questionnaires (15 for ages 2–4, 43 for ages 5–7, 71 for ages 8–12, and 39 for ages 13–18) and 104 PedsQL fatigue questionnaires (25 for ages 2–4, 28 for ages 5–7, 32 for ages 8–12, and 19 for ages 13–18). For PedsQL generic, age and sex of children suffering from chronic IF and the general population were not statistically different (*P* < 0.05).

PedsQL generic core scores for all age groups are presented in Table 2. The total score for ages 5 to 7 and ages 8 to 12 of children suffering from chronic IF was significantly lower than the general population with corresponding effect sizes of 0.73 and 0.71, respectively.

**TABLE 2:** Pediatric Quality of Life Inventory 4.0 generic score of patients suffering from chronic IF receiving home parenteral nutrition, compared with the general population

Age	PedsQL subscale	N	Score (IQR)	Norm, N = 293	*P* ^∗^	Effect size^∗∗^
2 to 4 years	Total	6	85 (79–90)	90	0.20	0.31
	Physical function		80 (77–98)	94	0.08	0.41
	Emotional function		83 (61–93)	80	0.90	0.03
	Social function		98 (75–100)	95	0.90	0.03
	School function		88 (73–100)	100	0.06	0.48
	Psychosocial function^		86 (77–94)	88	0.69	0.09

	PedsQL subscale	N	Score (IQR)	Norm (N = 274)	*P* ^∗^	Effect size^∗∗^
5 to 7 years	Total	15	**68 (55–77)**	**88**	**<0.01**	**0.73**
	Physical function		**50 (28–56)**	**94**	**<0.01**	**0.90**
	Emotional function		80 (60–90)	80	0.81	0.04
	Social function		**85 (60–95)**	**95**	**0.04**	**0.31**
	School function		**70 (65–95)**	**90**	**0.01**	**0.38**
	Psychosocial function^		**75 (63–87)**	**87**	**0.03**	**0.32**

	PedsQL subscale	N	Score (IQR)	Norm (N = 475)	*P* ^∗∗∗^	Effect size^∗∗^
8 to 12 years	Total	14	**68 (59–73)**	**87**	**<0.01**	**0.71**
	Physical function		**61 (31–77)**	**97**	**<0.01**	**0.89**
	Emotional function		75 (60–88)	80	0.31	0.16
	Social function		**75 (69–80)**	**90**	**<0.01**	**0.42**
	School function		**70 (61–85)**	**85**	**0.01**	**0.48**
	Psychosocial function^		**72 (64–81)**	**83**	**0.01**	**0.40**

	PedsQL subscale	N	Score (IQR)	Norm (N = 491)	*P* ^∗∗∗^	Effect size^∗∗^
13 to 18 years	Total	8	85 (77–92)	87	0.56	0.12
	Physical function		**78 (75–93)**	**97**	**<0.01**	**0.55**
	Emotional function		93 (71–100)	85	0.36	0.19
	Social function		90 (80–99)	90	0.96	0.01
	School function		83 (71–90)	80	0.66	0.09
	Psychosocial function^		88 (76–94)	83	0.56	0.12

All data are presented as median (interquartile range). Values highlighted in bold are considered statistically significantly different from normative data (18,19). IQR = interquartile range; PedsQL = Pediatric Quality of Life Inventory 4.0.

∗ Compared with Mann-Whitney *U* test to normative data (19).

∗∗ Calculated by methods described by Kerby (31).

∗∗∗ Compared with Mann-Whitney *U* test to normative data (18). ^Psychosocial function is the combined score of the emotional, social and school subscale.

PedsQL fatigue scores for all age groups are presented in Table 3. The total score for children suffering from chronic IF 5 to 7 years of age was significantly lower than normative data with an effect size of 0.70.

**TABLE 3:** Pediatric Quality of Life Inventory 4.0 fatigue score of patients suffering from chronic IF receiving home parenteral nutrition, compared with the general population

Age	PedsQL fatigue subscale	N	Score (IQR)	Norm	*P* ^∗^	Effect size^∗∗^
2 to 4 years	Total	17	78 (64–95)	83	0.18	0.34
	General fatigue		83 (54–94)	83	0.16	0.36
	Sleep		83 (50–96)	83	0.23	0.30
	Cognitive fatigue		83 (63–96)	83	0.30	0.26

	PedsQL fatigue subscale	N	Score (IQR)	Norm	*P* ^∗^	Effect size^∗∗^
5 to 7 years	Total	17	**75 (61–85)**	**83**	**0.01**	**0.70**
	General fatigue		**71 (50–75)**	**84**	**<0.01**	**0.81**
	Sleep		**75 (65–92)**	**88**	**0.02**	**0.64**
	Cognitive fatigue		67 (52–96)	77	0.34	0.24

	PedsQL fatigue subscale	N	Score (IQR)	Norm	*P* ^∗^	Effect size^∗∗^
8 to 12 years	Total	14	71 (63–83)	79	0.09	0.48
	General fatigue		73 (56–92)	83	0.06	0.55
	Sleep		73 (49–83)	78	0.08	0.51
	Cognitive fatigue		71 (65–82)	76	0.31	0.28

	PedsQL fatigue subscale	N	Score (IQR)	Norm	*P* ^∗^	Effect size^∗∗^
13 to 18 years	Total	8	75 (71–94)	75	0.41	0.31
	General fatigue		77 (65–100)	77	0.54	0.23
	Sleep		73 (56–90)	72	0.88	0.06
	Cognitive fatigue		83 (75–97)	77	0.11	0.64

All data are presented as median (interquartile range). Values highlighted in bold are considered statistically significantly different from normative data (20). IQR = interquartile range; PedsQL = Pediatric Quality of Life Inventory 4.0.

∗ Compared with 1-sample *t*-test to normative data (20).

∗∗ Calculated using Cohen d.

### Linear Mixed Models

**FIGURE 1:**
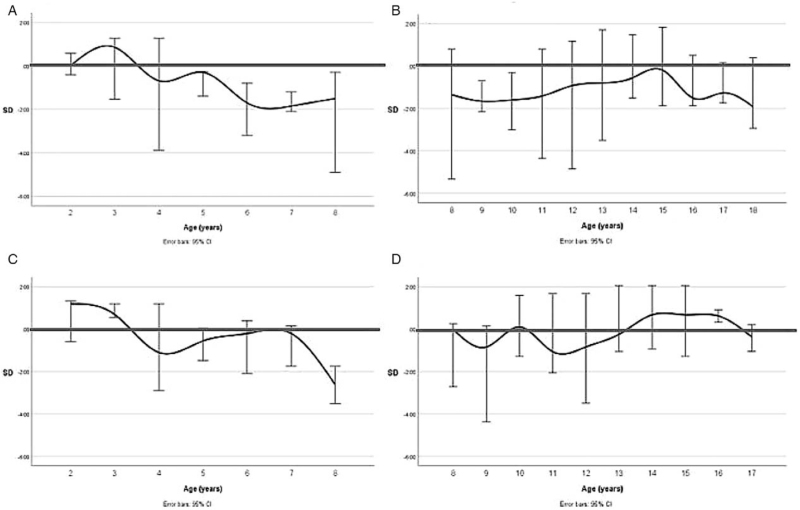
Course of Pediatric Quality of Life Inventory 4.0 generic total score SD for ages 2 to 7 years (proxy-report) and 8 to 18 years (self-report) (A and B, respectively) and PedsQL fatigue total score SD for ages 2 to 7 years and 8 to 18 years (C and D, respectively) over time of patients suffering from chronic intestinal failure. Bold line 0 SD indicates population mean. Error bars indicate a 95% confidence interval. PedsQL = Pediatric Quality of Life Inventory 4.0.

LMMs for PedsQL generic core total score for ages 2 to 7 and 8 to 18 years showed no statistical significant coefficient for duration of home PN; estimate −0.20 (95% CI: −0.44 to 0.05) and estimate 0.09 (95% CI: −0.02 to 0.19), respectively (Fig. 1). Also, underlying disease was not a statistically significant coefficient in the model for PedsQL generic core total score ages 2 to 7 or 8 to 18 years as depicted in Supplemental Digital Content 2.

LMMs for PedsQL fatigue total score ages 2 to 7 and 8 to 18 showed no statistical significant coefficient for duration of home PN; estimate −0.02 (95% CI: −0.35 to 0.30) and estimate 0.04 (95% CI: −0.09 to 0.18), respectively (Fig. 1). Prematurity was a statistically significant coefficient in the model for PedsQL fatigue total score ages 8 to 18 where children who were prematurely born scored significantly lower (estimated marginal mean −1.83SD) compared with those who were not (estimated marginal mean 0.04SD), *P* = 0.04. Underlying disease was not a statistically significant predictor in the model for PedsQL fatigue total score ages 2 to 7 or 8 to 18 as depicted in Supplemental Digital Content 2.

The number of night patients received PN per week was not a statistically significant coefficient in any model, as shown in Supplemental Digital Content 3.

## DISCUSSION

This is the first study assessing the longitudinal development of HRQOL in pediatric patients suffering from chronic IF. Patients ages 5 to 7 and 8 to 12 years scored significantly lower on the PedsQL generic core total score compared with the Dutch general population, with large effect sizes. In addition, patients ages 5 to 7 years scored significantly lower on the PedsQL fatigue total score compared with the Dutch general population. Patients, aged 2 to 4 and 13 to 18 years, reported comparable HRQOL to the Dutch general population. Surprisingly, this study showed that HRQOL does not significantly change over the duration of home PN therapy. Last, it is observed that for children ages 8 to 18 years, patients prematurely born had significantly greater fatigue than patients not prematurely born. Underlying disease or the number of nights per week on PN were not significantly associated with HRQOL in all age groups.

Several cross-sectional studies assessed long-term HRQOL in patients (that have been) dependent on PN. One study reported impaired HRQOL (measured with PedsQL generic core scale questionnaires) in patients (mean ages 12 years) with a history of IF who were not dependent on home PN at time of measurement (97% short bowel syndrome) (20). In contrast, others reported similar HRQOL for patients (mean age 9 years with 23% of patients receiving PN and 4 years with 100% of the patients receiving PN, respectively) with a history of IF when compared with healthy controls. In these cohort studies, 64% and 41% of patients suffered from short bowel syndrome, respectively.

Reasons for the lower HRQOL in patients ages 5 to 12 years, might be the distribution of underlying disease in our cohort differs from the majority of published studies since a lower proportion of patients suffering from short bowel syndrome and a higher proportion of patients suffering from intestinal neuromuscular motility disorders are included. We, however, did not find underlying disease to be associated with HRQOL. Also, our cohort differs from the majority of studies in that only patients who were dependent on PN were included. Median PN duration at last KLIK entry was 5.3 years, longer than all other studies (21).

A recent study on HRQOL in pediatric IF showed patients reported lower HRQOL scores than healthy controls. Interestingly, patients with IF scored similar HRQOL scores as patients suffering from other chronic gastrointestinal illnesses (22).

In our study, children ages 5 to 18 years scored significantly lower than the Dutch general population in the physical domain of the PedsQL generic score with medium-to-large effect sizes. These findings, which are in agreement with previous studies, reflect the large impact pediatric chronic IF has on patients’ physical functioning. In all cross-sectional studies, impaired physical functioning was reported when compared with a reference group or score (20,23–28).

One recently published study on fatigue in children suffering from pediatric IF reported greater fatigue in 21 patients (median age 8.3 years) when compared with healthy controls (9). This was also partly observed in our study. Children ages 5 to 7 years scored significantly lower than the Dutch general population with regards to fatigue. We did not observe greater fatigue in patients ages 8 to 12 years, which could be because of the small sample size.

With LMMs, the evolution of HRQOL over long-term treatment with home PN was assessed, no significant change was observed for both the PedsQL generic core scale and the PedsQL fatigue scale. This is the first study that assessed the longitudinal development of HRQOL during home PN treatment. Neam et al annually measured proxy HRQOL in patients with (a history of) IF. Development of HRQOL over time is not reported in this study, possibly because of the fact that 44 of the 91 participants completed only 1 questionnaire hampering the ability of the researchers to adequately assess development of HRQOL (29). In a study concerning childhood cancer patients who were recruited during or in the first year after treatment, PedsQL fatigue score reported by patients was worse than Dutch norm scores at baseline and 4 months but recovered at 12 months (30). Our results do not show a normalization of HRQOL over time. This might be because of the fact that the childhood cancer patients were followed during remission, compared with our cohort who continue to be actively treated. Assessment of HRQOL and fatigue after weaning from PN might provide valuable insights on this and should be performed in future studies.

In our study, children who were prematurely born reported significantly lower PedsQL fatigue scores when of age 8 to 18 years. This was not reported in other studies on HRQOL or fatigue (9,24,25). Neam et al (29) reported that patients with developmental delay, which often occurs in prematurely born children, were independently associated with lower HRQOL scores but did not report on whether prematurely born children had lower HRQOL. Cognitive fatigue is one of the measured subscales in the PedsQL fatigue questionnaire, our finding, therefore, might in part reflect the effect of premature birth on HRQOL fatigue.

Underlying disease or the number of days per week patients received PN did not show a statistical association with HRQOL in our cohort. This was also not observed in cross-sectional studies on HRQOL in pediatric IF in which the association between HRQOL and these factors was analyzed (23–25). In adults, however, who completed the parenteral nutrition impact questionnaire, underlying disease explained differences in HRQOL, in which patients suffering from severe gastrointestinal dysmotility reported a higher HRQOL than patients with other diagnoses, such as short bowel syndrome (31). Reason for not finding an association between underlying disease and HRQOL could be our sample size. A reason for not finding an association between the number of nights patients received PN and HRQOL and fatigue might be because of the limited distribution of this associative parameter with most patients receiving PN for 6 to 7 nights per week.

Adult patients who required a greater number of nights of home PN per week showed worse HRQOL scores (31). Reason for not finding an association between number of nights on home PN and HRQOL in our cohort might be the fact that the majority of patients is cared for by their parents at home and is still able to attend all social activities with their friends. For example, parents enable their children to sleep over at friends by transporting all infusion equipment to the other house and being “on call” in case anything happens overnight during the infusion of PN. The efforts by parents to facilitate an as normal as possible childhood for their chronically ill child are laudatory. Structural screening for parental psychosocial problems is warranted as home PN treatment of a child is highly stressful and influences daily functioning (4).

Limitations of this study are the relatively low number of included patients, a common fact in studies concerning pediatric IF indicated by the fact that this is the third largest cohort of children suffering from chronic IF in which HRQOL is studied. Furthermore, our participation rate of 69% may imply selection bias as parents or patients with decreased HRQOL and or increased fatigue may be more inclined to participate in the KLIK project. Future studies should also assess if duration of daily PN infusion (hours per day) might influence these outcomes. Furthermore, international collaboration is vital in future studies as to compare the effect of different therapeutic approaches between countries on HRQOL and fatigue and to take into account the possible effect of different cultural backgrounds on HRQOL and fatigue.

## CONCLUSIONS

Children with chronic IF on home PN 5 to 12 years of age report lower HRQOL scores compared with the Dutch general population. HRQOL does not change during long-term treatment with HPN in children suffering from chronic IF, which could be because of the symptoms of the disease and the burden of PN treatment. Structural monitoring of relevant patient outcomes in clinical practice is warranted in these patients, to identify and discuss problems, and offer tailor-made interventions.

## Supplementary Material

Supplemental Digital Content
