# Supplementary material for: Longitudinal Development of Health-related Quality of Life and Fatigue in Children on Home Parenteral Nutrition
Source: J Pediatr Gastroenterol Nutr. 2021 Oct 22;74(1):116–22. doi: 10.1097/MPG.0000000000003329 (PMC8673843; doi:10.1097/MPG.0000000000003329)
Supplement: Supplemental Digital Content [file jpga-74-116-s001.pdf]

Supplemental digital content 1.

Pediatric Quality of Life Inventory 4.0 (PedsQL) Generic

The PedsQL is a 23 item questionnaire assessing HRQOL using a one-week recall period. If a child was 2 to 7 years of age, parents completed the generic PedsQL, proxy toddler version (2-4 years) or proxy young child version (5-7 years). If patients were 8 years or older and were deemed able to complete the questionnaires themselves, they completed the PedsQL child version for children aged 8-12 years or the adolescent version for children aged 13-18 years.

All questions are answered on a five point Likert scale and grouped in 4 scales: physical domain, emotional domain, social domain and school domain. The 4 scales are grouped under a total score and a psychosocial score (combined score of emotional, social and school domain). All scales are scored from 0-100; higher scores indicate better HRQOL. The reliability and validity of the questionnaire have been demonstrated(11, 12).

Previous research has shown that reliability and validity of the PedsQL is good(12, 13). For the PedsQL proxy report and self-report versions, normative data of the Dutch general population was used(14, 15) (15).

PedsQL fatigue

The PedsQL fatigue is an 18 item questionnaire assessing complaints and burden of fatigue using a one-week recall period. The PedsQL fatigue questionnaire was completed (proxy report: child 2-7 years, self: child ≥ 8). All questions are answered on a five point Likert scale and grouped in three scales: general fatigue, sleep/rest fatigue and cognitive fatigue. All scales are scored from 0-100; higher scores indicate fewer fatigue symptoms. This questionnaire demonstrated adequate reliability and validity(16, 17). For the PedsQL fatigue questionnaire, normative data of the Dutch general population were used(16).

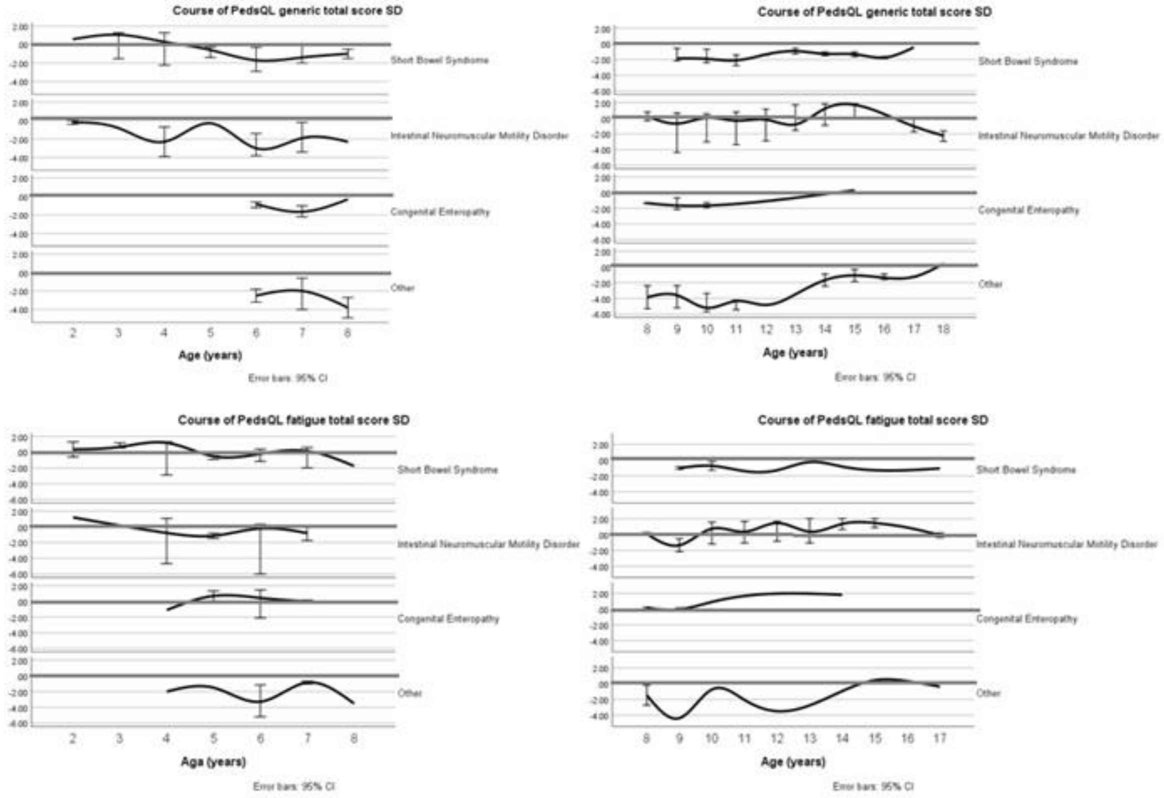

Figure S1. Course of PedsQL generic total score SD for ages 2-7 and 8-18 years (A & B, respectively) and PedsQL fatigue total score SD for ages 2-7 and 8-18 years (C & D, respectively) over time of patients suffering from chronic intestinal failure, grouped per underlying disorder. Patients suffering from short bowel syndrome, intestinal neuromuscular motility disorders or congenital enteropathies show a higher HRQOL when compared to children suffering from other underlying conditions. This difference does not reach statistical significance in any of the four models.

Bold line 0 SD indicates population mean.

Error bars indicate a 95% confidence interval.

Supplementary table 1. summaries of linear mixed models for PedsQL generic core total score and PedsQL fatigue total score.

**PedsQL generic core total score ages 2-7 years** (58 observations in 19 patients)			
**Parameter**	**Estimate**	**95% Confidence interval**	**P**
**(Intercept)**	-1.37	-4.20 – 1.47	.33
**Premature (no)**	.37	-.84 – 1.57	.52
**Underlying diagnosis (short bowel syndrome)**	1.57	-.32 – 3.45	.20
**Underlying diagnosis (motility disorder)**	.46	-1.49 – 2.42	
**Underlying diagnosis (congenital enteropathy)**	1.50	-.70 – 3.69	
**Duration of home PN**	-.13	-.39 – .13	.31
**Days PN per week**	-.09	-.32 – .13	.39
**PedsQL generic core total score ages 8-18 years** (110 observations in 18 patients)			
**Parameter**	**Estimate**	**95% Confidence interval**	**P**
**(Intercept)**	-4.50	-6.60 – -2.39	**.01**
**Premature (no)**	1.90	-.16 – 3.97	.07
**Underlying diagnosis (short bowel syndrome)**	1.00	-1.35 – 3.34	.18
**Underlying diagnosis (motility disorder)**	1.80	-.15 – 3.73	
**Underlying diagnosis (congenital enteropathy)**	1.91	-.16 – 3.97	
**Duration of home PN**	.08	-.01 – .17	.07
**Days PN per week**	-.06	-.22 – .10	.48

**PedsQL fatigue total score ages 2-7 years** (53 observations in 25 patients)			
**Parameter**	**Estimate**	**95% Confidence interval**	**P**
**(Intercept)**	-1.80	-5.08 – 1.49	.27
**Premature (no)**	-.25	-1.58 – 1.09	.70
**Underlying diagnosis (short bowel syndrome)**	1.93	-.20 – 4.06	.24
**Underlying diagnosis (motility disorder)**	1.65	-.49 – 3.80	
**Underlying diagnosis (congenital enteropathy)**	2.08	-.28 – 4.44	
**Duration of home PN**	.03	-.27 – .33	.84
**Days PN per week**	-.08	-.34 – .19	.56
**PedsQL fatigue total score ages 8-18 years** (51 observations in 18 patients)			
**Parameter**	**Estimate**	**95% Confidence interval**	**P**
**(Intercept)**	-2.72	-4.71 – -.73	**.01**
**Premature (no)**	1.86	.09 – 3.64	**.04**
**Underlying diagnosis (short bowel syndrome)**	.06	-2.03 – 2.15	.08
**Underlying diagnosis (motility disorder)**	.93	-.73 – 2.60	
**Underlying diagnosis (congenital enteropathy)**	2.16	.39 – 3.94	
**Duration of home PN**	.02	-.10 – .15	.72
**Days PN per week**	-.01	-.17 – .14	.86

Intraclass Correlation Coefficients for intercept are: .13 for PedsQL generic 2-7, .68 for PedsQL generic 8-18, .30 for PedsQL fatigue 2-7 and .41 for PedsQL fatigue 8-18

PN: parenteral nutrition

Intercept: expected mean SD value if all parameters are coded zero.
